# Statins Stimulate New Myocyte Formation After Myocardial Infarction by Activating Growth and Differentiation of the Endogenous Cardiac Stem Cells

**DOI:** 10.3390/ijms21217927

**Published:** 2020-10-26

**Authors:** Eleonora Cianflone, Donato Cappetta, Teresa Mancuso, Jolanda Sabatino, Fabiola Marino, Mariangela Scalise, Michele Albanese, Alessandro Salatino, Elvira Immacolata Parrotta, Giovanni Cuda, Antonella De Angelis, Liberato Berrino, Francesco Rossi, Bernardo Nadal-Ginard, Daniele Torella, Konrad Urbanek

**Affiliations:** 1 Department of Medical and Surgical Sciences, Magna Graecia University, 88100 Catanzaro, Italy; cianflone@unicz.it (E.C.); jolesbt@hotmail.it (J.S.); mikelealbanese@gmail.com (M.A.); parrotta@unicz.it (E.I.P.); bernardo.nadalginard@gmail.com (B.N.-G.); 2 Department of Experimental Medicine, University of Campania “L. Vanvitelli”, 80138 Naples, Italy; donato.cappetta@unicampania.it (D.C.); antonella.deangelis@unicampania.it (A.D.A.); liberato.berrino@unicampania.it (L.B.); francesco.rossi@unicampania.it (F.R.); 3 Department of Experimental and Clinical Medicine, Magna Graecia University, 88100 Catanzaro, Italy; tmancuso@unicz.it (T.M.); marino@unicz.it (F.M.); m.scalise@unicz.it (M.S.); salatino@unicz.it (A.S.); cuda@unicz.it (G.C.)

**Keywords:** Statins, 3-hydroxy-3-methylglutaryl coenzyme A, cardiac stem cells, myocardial regeneration, Akt

## Abstract

The 3-hydroxy-3-methylglutaryl coenzyme A (HMG-CoA) reductase inhibitors (statins) exert pleiotropic effects on cardiac cell biology which are not yet fully understood. Here we tested whether statin treatment affects resident endogenous cardiac stem/progenitor cell (CSC) activation in vitro and in vivo after myocardial infarction (MI). Statins (Rosuvastatin, Simvastatin and Pravastatin) significantly increased CSC expansion in vitro as measured by both BrdU incorporation and cell growth curve. Additionally, statins increased CSC clonal expansion and cardiosphere formation. The effects of statins on CSC growth and differentiation depended on Akt phosphorylation. Twenty-eight days after myocardial infarction by permanent coronary ligation in rats, the number of endogenous CSCs in the infarct border zone was significantly increased by Rosuvastatin-treatment as compared to untreated controls. Additionally, commitment of the activated CSCs into the myogenic lineage (c-kit^pos^/Gata4^pos^ CSCs) was increased by Rosuvastatin administration. Accordingly, Rosuvastatin fostered new cardiomyocyte formation after MI. Finally, Rosuvastatin treatment reversed the cardiomyogenic defects of CSCs in c-kit haploinsufficient mice, increasing new cardiomyocyte formation by endogenous CSCs in these mice after myocardial infarction. In summary, statins, by sustaining Akt activation, foster CSC growth and differentiation in vitro and in vivo. The activation and differentiation of the endogenous CSC pool and consequent new myocyte formation by statins improve myocardial remodeling after coronary occlusion in rodents. Similar effects might contribute to the beneficial effects of statins on human cardiovascular diseases.

## 1. Introduction

The demonstration that new cardiomyocytes (CMs) are produced in the adult mammalian myocardium, albeit in limited amounts, generated a flurry of interest in harnessing this adult neo-cardiomyogensis to foster adult myocardial regeneration and repair in order to ameliorate the devastating impact of myocardial damage and heart failure in an aging human population [1,2,3]. This paradigmatic change, together with the identification and characterization of adult cardiac stem cells (CSCs) [1] gave birth to the bourgeoning field of adult myocardial regeneration. Unfortunately, since its inception, myocardial regeneration in the adult has been hotly debated and controversial. While the discovery and characterization of the phenotype and regenerative potential of adult resident cardiac stem/progenitor cells was initially met as a significant advancement in cardiac biology, some recent data from genetic fate mapping studies have questioned the endogenous regenerative role of CSCs after myocardial infarction [4,5,6,7,8,9]. However, these latter data remain doubtful because the technology used to purportedly track the fate of the CSCs in vivo fails to efficiently label and track them [4,5,6,7,8,9].

Aside from the ongoing controversy, several studies have shown that CSCs, apart from their cardiomyocyte regenerative potential, are promising regenerative agents for their paracrine action when used as allogenic cell therapy [10,11,12,13,14,15,16,17] and their regenerative potential can be fostered by several experimental approaches [3,10,11,12,13,18,19,20,21]. HMG-CoA reductase inhibitors (statins) are well known lipid-lowering drugs that significantly and reproducibly reduce morbidity and mortality from both acute and chronic coronary syndromes [22,23]. Besides their primary lipid-lowering effect, statins exert pleiotropic beneficial effects on the cardiovascular system in primary and secondary prevention: they improve cardiomyocyte survival [24], reduce vascular inflammation [25,26] and decrease platelet aggregation and thrombus deposition [27,28]. Of interest, statins have been shown to affect the biology and function of several stem and progenitor cells, improving their regenerative potential and preventing their age-dependent senescent phenotype [29,30,31,32]. On this basis, to gain further insights into the mechanisms by which statins improve myocardial remodeling, here we investigated the effects of statins on resident CSCs in vitro and in vivo.

## 2. Results

### 2.1. HMG-CoA Reductase Inhibitors Enhance Rat Cardiac Stem Cell Growth, Clonogenesis and Spherogenesis In Vitro

**Figure 1:**
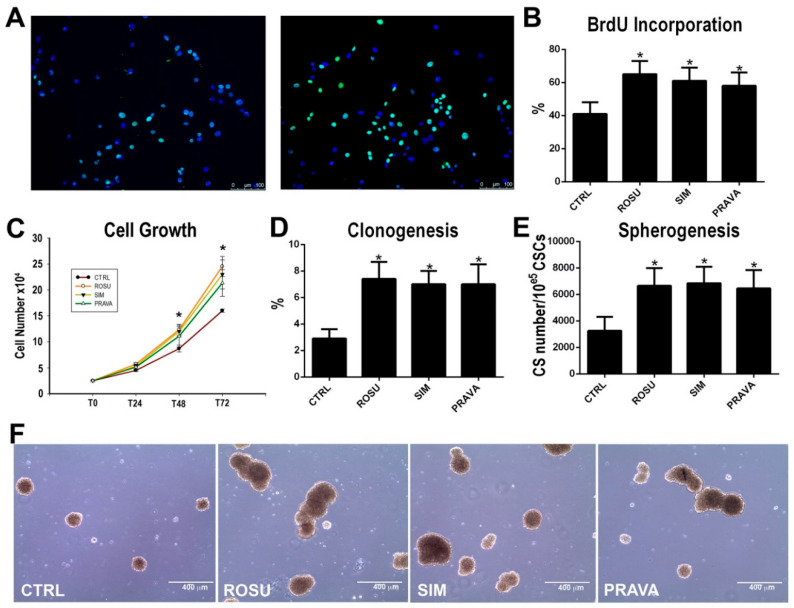
The Effects of HMG-CoA reductase inhibitors on rat cardiac stem cell growth in vitro. (**A–C**) Effects of Rosuvastatin (ROSU, 1 µM), Simvastain (SIM, 1 µM) or Pravastatin (PRAVA, 5 µM) on CSC proliferation, respectively, evaluated by BrdU incorporation and growth curve at the indicated time points. * *p* < 0.05 vs. CTRL. Data are Mean ± SD, *n* = 6. (**D**) CSCs show a significant higher clonogenic potential when treated with Rosuvastatin (ROSU, 1 µM), Simvastain (SIM, 1 µM) or Pravastatin (PRAVA, 5 µM) as compared to untreated control cells (CTRL). * *p* < 0.05 vs. CTRL. Data are Mean ± SD, *n* = 6. (**E**) CSCs show a significant higher spherogenic potential (CardioSphere -CS- number) when treated with Rosuvastatin (ROSU, 1 µM), Simvastain (SIM, 1 µM) or Pravastatin (PRAVA, 5 µM) as compared to untreated control cells (CTRL). * *p* < 0.05 vs. CTRL. (**F**) Representative images of CSC-derived cardiospheres are shown in the presence or absence of SIM where number and size of spheres are increased by statins, bar = 50 μm. Data are Mean ± SD, *n* = 6.

To test whether HMG-CoA reductase inhibitors (statins) regulate adult CSC proliferation in vitro, BrdU incorporation and cell growth curve were employed. To this aim we isolated CD45^neg^/CD31^neg^/c-kit^pos^ CSC-enriched cardiac cells (hereafter abbreviated as CSCs) from rat donors as previously reported [11,12,33] and plated them in presence or absence of a lipophilic or hydrophilic statin (see below). To assess the effects of statins on the rat CSC cell cycle activation in vitro, primary cultured CSCs at P1 were serum starved for 48 h and parallel cultures were then treated either with 1 µM simvastatin (SIM), as a prototypical lipophilic HMG-CoA reductase inhibitor, 1 µM rosuvastatin (ROSU) or 5 µM pravastatin (PRAVA), respectively, as new and old generation hydrophilic HMG-CoA reductase inhibitors, or just vehicle. BrdU (10 µM) was added every 12 h and BrdU incorporation was measured after 24 h by immunostaining. Figure 1 shows that ROSU, SIM and PRAVA treatment increased on average the number of BrdU positive CSCs by 50% when compared to vehicle (Figure 1A,B). Concurrently, and in agreement with the BrdU data, the growth curve shows that by 72 h in culture, ROSU, SIM and PRAVA on average increased the number of CSCs by 55% when compared to vehicle (Figure 1C).

We then evaluated the effects of ROSU treatment on CSC clonal formation in vitro by single cell deposition. From a total of 1301 single CSCs seeded at P1 in clonogenic medium in 96-well plates, clones were detected in 38 wells at 14 days (Figure 1D). ROSU, SIM and PRAVA treatment significantly increased number of clones: from a total of 1310, 1322 and 1305 single deposited CSCs, respectively, 97, 103 and 91 clones were retrieved after 14 days, reflecting on average an almost three-fold increase over the drug-free cell control (Figure 1D).

Finally, we tested the effects of ROSU, SIM and PRAVA on spherogenesis, a typical feature of multipotent stem cells [11,12]. When CSCs at P1 were grown unattached in air with 3% O_2_, they formed cardiospheres (CS) at a rate of 3250 ± 1050 per 10^5^ cells (Figure 1E,F). ROSU, SIM and PRAVA significantly increased the number of CSC-derived CS to 6650 ± 1350, 6850 ± 1250 and 6450 ± 1400 respectively, per 10^5^ cells, which represents a two-fold increase, closely reproducing the effects of statins on clonogenesis (Figure 1E,F).

Overall, these data show that HMG-CoA reductase inhibitors foster CSC activation by enhancing their growth and robustly boost their clonogenesis and spherogenesis in vitro.

### 2.2. HMG-CoA Reductase Inhibitors Activate the Protein Kinase Akt and Promote CSC Survival In Vitro

Previous data have shown that Akt phosphorylation is one of the main molecular target of HMG-CoA reductase inhibitors in stem and progenitor cells [31]. Thus, to test whether the beneficial effects of statins on cell activation is mediated by the Akt signaling pathway, CSCs were incubated with HMG-CoA reductase inhibitors and Akt phosphorylation was assessed through western blot analysis in short- and long-term cell cultures.

**Figure 2:**
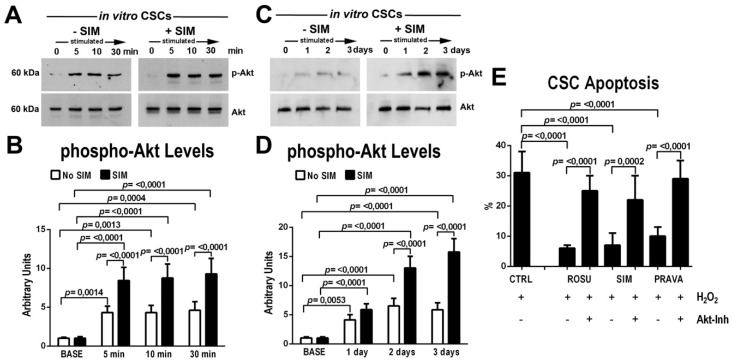
HMG-CoA reductase inhibitors activate the protein kinase Akt and promote CSC survival in vitro. (**A,B**) Representative western blot analysis and cumulative data showing that Simvastatin (SIM, 1 µM) increases Akt phosphorylation (Ser-473) in CSCs in vitro in the short time course analysis when compared to placebo-treated control cells (BASE). Phospho-Akt densitometry value was normalized to total Akt (pAkt/Akt ratio). Representative of four biological replicates. (**C,D**) Representative western blot analysis and cumulative data showing that daily Simvastatin administration (SIM, 1 µM) increases Akt phosphorylation in CSCs in vitro in a 3-day analysis when compared to placebo-treated control cells (BASE). pAkt levels were expressed as pAkt/Akt ratio as above. Representative of four biological replicates. (**E**) CSCs show a significant decreased H_2_O_2_-induced apoptotic death (TdT + cells) at 8h when treated with Rosuvastatin (ROSU, 1 µM), Simvastain (SIM, 1 µM) or Pravastatin (PRAVA, 5 µM) as compared to untreated control cells (CTRL). The Akt inhibitor (MK2206, 1 µM) prevented the beneficial effects of statins in vitro. Representative of four biological replicates. All data are Mean ± SD.

Simvastatin treatment led to a significant increase (two fold on average at each time point) on top of 5% serum in Akt phosphorylation from 5 through 30 min (Figure 2A,B) when compared to control vehicle treated cells (Figure 2A,B). When CSCs were daily cultured in 10% serum in presence or absence of SIM, Akt phosphorylation was at each time point higher in SIM-treated CSCs versus untreated CSCs (Figure 2C,D). Similar data were obtained treating CSCs with Rosuvastatin and Pravastatin (data not shown).

Considering the main role of Akt in cell survival [34], we tested whether statins decrease CSC death when under oxidative stress conditions by H_2_O_2_ administration. As expected, H_2_O_2_ induced significant apoptosis (measured by TdT assay) in more than 30% of treated CSCs in vitro (Figure 2E). SIM, PRAVA and ROSU administration decreased the apoptotic death of H_2_O_2_-damaged CSCs approximately four-fold (Figure 2E). Importantly, selective Akt blockage by a specific Akt inhibitor, MK2206, practically abolished the beneficial effects of statins on CSC survival (Figure 2E).

### 2.3. HMG-CoA Reductase Inhibitors Foster Myogenic Commitment of CSCs In Vitro

**Figure 3:**
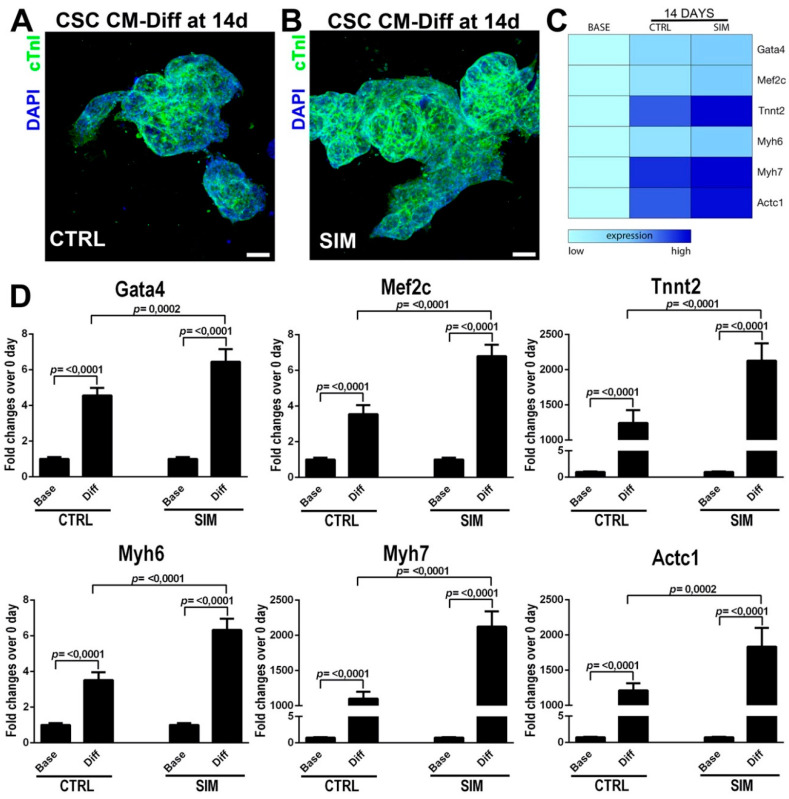
HMG-CoA reductase inhibitors foster myogenic commitment of CSCs in vitro. (**A,B**) Confocal images of cardiac Troponin I (cTnI) expression in cardiospheres from CSCs in vitro in presence or absence of SIM (1 µM) after 14 days in myogenic media (representative of *n* = 5 biological replicates). Scale bar = 75 µm. (**C**) Heatmap of RT-PCR analysis of main linage-specific transcription factors and contractile markers in cardiospheres from CSCs in presence or absence of SIM (1 µM) after 14 days in myogenic media (representative of *n* = 3 number of biological replicates). (**D**) mRNA levels of the myogenic transcription factors and contractile genes after myogenic differentiation of CSCs treated with SIM. SIM significantly upregulated the mRNA levels of GATA-4, MEF2C, cTNNT2, alpha- and beta-MHC and cardiac actin. Levels of each mRNA target was first normalized to GAPDH mRNA level and then transformed as fold change from base (0 day), which was by default set at 1. All data are Mean ± SD.

We then investigated the effects of HMG-CoA reductase inhibitors on CSC myogenic differentiation. Myogenic differentiation in rat CSCs was induced as described previously [11,12], in absence or presence of SIM (1 µM) treatment for 14 days and then stained treated cells for myocyte lineage-specific markers (Figure 3A,B).

SIM administration lead to an increase in the number of CSC-derived cTnI+ cardiomyocytes in differentiating cardiospheres (83 ± 8 vs. 65 ± 7 in control untreated cells, *p* < 0.05) (Figure 3A,B). To further assess the effect of SIM on CSC myogenic differentiation, we measured the expression of linage-specific transcription factors and contractile markers using real-time PCR. mRNA levels of the myogenic transcription factor Gata4 and MEF2C were significantly increased (~doubled vs. control) upon differentiation of CSCs treated with SIM (Figure 3C,D). Furthermore, SIM significantly upregulated (~doubled vs. control) the mRNA levels of the contractile genes cTNNT2, alpha- and beta-MHC and cardiac actin (Figure 3C,D). Similar results were obtained using both ROSU and PRAVA (data not shown).

These data indicate that statins influence lineage specification of CSCs fostering cardiomyocyte commitment of these cells in culture.

### 2.4. HMG-CoA Reductase Inhibitors Increase CSC Number and New Myocyte Formation After Myocardial Infarction

**Figure 4:**
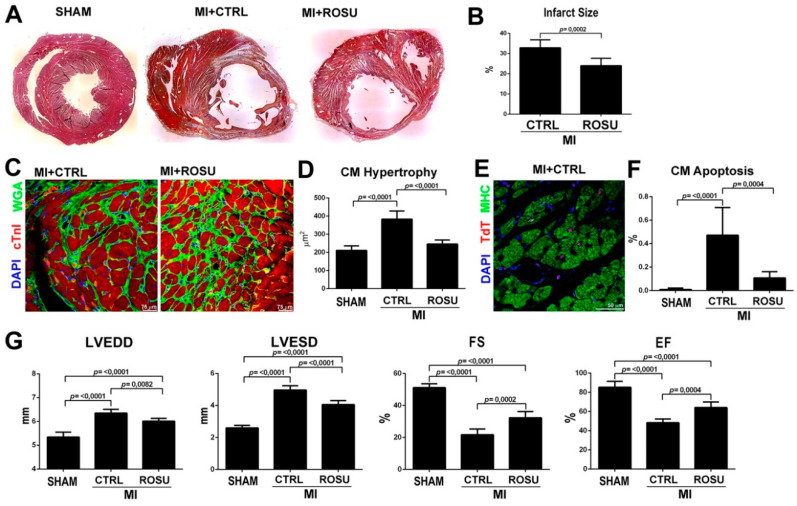
Rosuvastatin improves myocardial remodeling after myocardial infarction in rats. (**A**) Representative Hematoxylin & Eosin (H&E) staining of rat cardiac cross sections at the mid/apex from Sham operated, placebo control and Rosuvastatin (ROSU) treated rats 28 days after MI. It is evident that myocardial scarring was reduced by ROSU when compared to placebo control MI. (**B**) Bar graph showing that ROSU significantly reduced infarct size 28 days after MI. (**C,D**) Representative rat cardiac cross-sections (WGA, green; troponin, cTnI, red; DAPI, blue nuclei) and bar graph with cumulative data show CM size and hypertrophy. Placebo control-treated rats show significant CM hypertrophy 28 days after MI when compared to sham operated rats. ROSU prevented CM hypertrophy; * *p* < 0.05 vs. all. (**E**) Representative confocal image shows cardiomyocyte apoptosis (TdT staining, red) in placebo control treated animals 28 days after MI. (**F**) Bar graph with cumulative data show that treatment with ROSU significantly reduced cardiomyocyte apoptosis 28 days after MI when compared to placebo control treated rats (MI); * *p* < 0.05 vs. SHAM; # *p* < 0.05 vs. MI. (**G**) Bar graph showing cumulative echocardiographic data on LV end-diastolic diameter (LVEDD), LV end-sistolic diameter (LVESD), fractional shortening (FS) and ejection fraction (EF) in the different groups included in the study. * *p* < 0.05 vs. sham; and # *p* < 0.05 vs. placebo control treated rats (MI). Data are Mean ± SD. SHAM, *n* = 5; MI (placebo control), *n* = 5; MI + ROSU, *n* = 6.

On the basis of the in vitro data shown above, we endeavored to explore whether the HMG-CoA reductase inhibitors modify CSC activation and new myocyte formation after myocardial infarction in rats. Because the in vitro data were reproducible using both hydrophilic and lipophilic HMG-CoA reductase inhibitors, the following experiments were performed using ROSU simply because this is the last generation of this drug class clinically-introduced. Female Wistar rats were administered with ROSU (20 mg/Kg daily) in 8 mL of drinking water (when this water was finished, the animals had access to drinking water ad libitum every day) for two weeks before and 4 weeks after permanent ligation of the left anterior descendent (LAD) coronary artery when the animals were sacrificed. Control rats received drinking water only before and after LAD ligation. The cardiac tissue regenerative potential of ROSU was assessed by subcutaneously implanting, right after LAD ligation procedure, osmotic mini-pumps to systemically release BrdU in order to label and track new cardiac cell formation [11]. Twenty-eight days after LAD ligation, in agreement with previous data on statins effects on post-MI myocardial remodeling [35], ROSU significantly decreased infarct scar size by ~30% (24 ± 4% vs. 33 ± 4% in CTRL untreated MI rats, *p* = 0.0002), preventing reactive cardiomyocyte hypertrophy (246 ± 23 µm^2^ vs. 384 ± 45 µm^2^ in CTRL untreated MI rats, *p* < 0.0001) and reducing by ~80% cardiomyocyte apoptosis (0.11 ± 0.05% vs. 0.47 ± 0.24% in CTRL untreated MI rats, *p* = 0.0004) (Figure 4A–F).

The latter beneficial anatomical and tissue effects of ROSU on post-MI remodeling ensued in decreased LV end-diastolic (6.01 ± 0.12 mm vs. 6.35 ± 0.16 mm, *p* = 0.0082) and end-systolic (4.05 ± 0.25 mm vs. 4.97 ± 0.27 mm, *p* < 0.0001) dimensions (Figure 4G) when compared to untreated MI control rats, suggesting a more pronounced effects of ROSU on systolic than diastolic LV function. Consequently, ROSU improved LV fractional shortening (32.3 ± 3.9% vs. 21.7 ± 3.5%, *p* = 0.0002) and LV ejection fraction (64.1 ± 5.9% vs. 48.3 ± 3.8%, *p* = 0.0004) compared to untreated MI control rats (Figure 4G).

**Figure 5:**
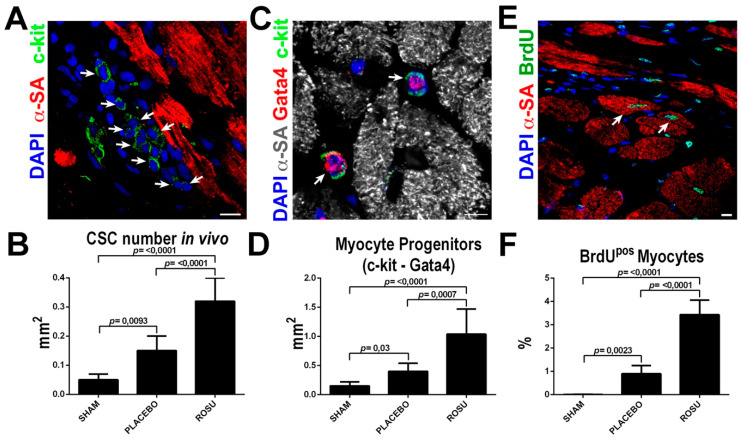
The HMG-CoA reductase inhibitors increase CSC number and new myocyte formation after myocardial infarction in vivo. (**A,B**) Representative confocal image and bar graph show an increased number of uncommitted lineage negative CSCs (arrows) in the border infarct zone in ROSU treated animals when compared to untreated counterparts. Scale Bar = 10 μm. (**C,D**) Representative confocal image and bar graph show an increased number of CSCs with myogenic commitment (myocyte progenitors) in ROSU treated animals as revealed by Gata4 expression (arrows). Scale Bar = 10 μm. (**E**) Representative confocal microscopy image of BrdU incorporation (BrdU positive, green fluorescence, arrows) in the border zone of a ROSU-treated infarcted rat heart. Scale bar 50 μm. (**F**) Number of newly-generated BrdU^pos^ cardiomyocytes 28 days after MI in rats untreated (placebo control, CTRL) or treated with ROSU; * *p* < 0.05 vs. all *n* = 6.

More importantly, ROSU increased ~two-fold the number of uncommitted lineage negative CSCs (identified as previously described [13]) in the border infarct zone when compared to untreated animals (Figure 5A,B). Concurrently, ROSU fostered the specification of CSCs towards the myogenic lineage as revealed by a ~3 fold increase of Gata4 expressing CSC-committed myocyte precursors (Figure 5C,D). The latter was accordingly associated with a significant ~3-fold increase of newly formed BrdU positive cardiomyocytes in the border zone of ROSU treated vs. untreated rats (0.9 ± 0.35% vs. 3.4 ± 0.63%, respectively, *p* < 0.0001) (Figure 5E,F).

Altogether, these data confirm that HMG-CoA reductase inhibitors favorably affect cardiac remodeling after myocardial infarction and, for the first time, show that the improved myocardial response upon ischemic/necrotic insult by statins also encompasses a boost in resident CSC activation, ensuing in significantly higher new myocyte formation. However, these data did not address the effects of statins on other aspects of myocardial tissue repair and regeneration after MI, such as neo-angiogenesis and fibrosis, which has been nonetheless already evaluated in previous reports [23,24].

### 2.5. HMG-CoA Reductase Inhibitors Ameliorate the In Vitro and In Vivo Regenerative Defect of CSCs from c-Kit Haploinsufficient Mice

**Figure 6:**
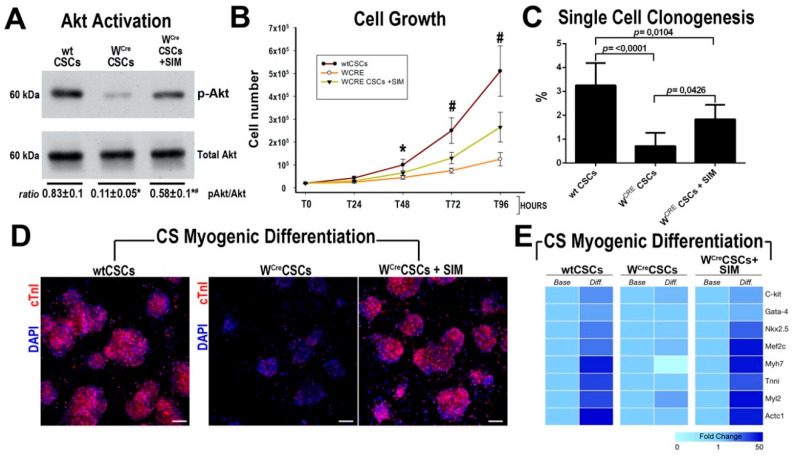
HMG-CoA reductase inhibitors ameliorate in vitro regenerative defect of CSCs from c-kit haploinsufficient mice. (**A**) Representative western blot analysis and densitometry cumulative analysis show that phospho-Akt (Ser-473) levels in response to serum (10%) in culture are significantly reduced in W^Cre^CSCs when compared to wtCSCs. SIM treatment rescues the levels of Akt phosphorylation in W^Cre^CSCs. * *p* < 0.05 vs. wtCSCs, # *p* < 0.05 vs. W^Cre^CSCs. Representative of four biological replicates. (**B**) Cell growth curve in culture over the indicated time points of wtCSCs vs. W^Cre^CSCs in the presence or absence of 1 μM SIM (Representative of n = 6 number of biological replicates). (**C**) Clonal efficiency wtCSCs vs. W^Cre^CSCs in presence or absence of 1 μM SIM (Representative of n = 6 number of biological replicates). (**D**) Confocal images of cardiac Troponin I (cTnI) expression in cardiospheres from wtCSCs and W^Cre^CSCs and W^Cre^ CSCs in presence or absence of SIM (1μM) after 14 days in myogenic media. (Representative of *n* = 5 biological replicates). Scale bar = 75 µm. (**E**) Heatmap of RT-PCR analysis of main cardiomyocyte genes modulation in cardiospheres from wtCSCs and W^Cre^CSCs and W^Cre^ CSCs in presence or absence of SIM (1 μM) after 14 days in myogenic media (Representative of *n* = 3 number of biological replicates). All data are Mean ± SD.

We have recently shown that c-kit/CRE mice generated by Cre insertion at the ATG of the Exon 1 of the c-kit locus produce a c-kit haploinsufficiency and fail to fate map c-kit-expressing CSCs and are, therefore, unable to correctly establish CSC contribution to new cardiomyocytes in vivo [10]. Furthermore, c-kit haploinsufficiency inhibits the regenerative potential of c-kit expressing CSCs [10,13]. Importantly, c-kit kinase receptor dependent molecular cascade rolls down to MAPK and Akt activation [36] and CSCs from c-kit haploinsufficient mice show a reduced Akt activation in cell culture in response to serum (10%) (Figure 6A).

Thus, we tested whether HMG-CoA reductase inhibitors could rescue/improve the in vitro regenerative biology of CSCs isolated from c-kit haploinsufficient c-kit^CreER/T2^ mice (hereafter, W^Cre^CSCs where W identifies the typical White mutation created by Cre insertion in the c-kit locus making a null c-kit allele) [10,13,37,38,39]. SIM administration (1 μM) increased AKT phosphorylation in W^Cre^CSCs when compared to untreated cells (Figure 6A). Accordingly, SIM increased W^Cre^CSCs proliferation and clonal formation, which still show an incomplete rescue of their expansion capacity when compared to wt CSCs (Figure 6B,C). W^Cre^ CSCs poorly differentiated into cTnI positive myocytes in vitro when compared to wt CSCs (Figure 6D). Accordingly, cardiac-specific gene transcripts were minimally upregulated compared to relative transcript levels in undifferentiated W^Cre^ CSCs (Figure 6E). SIM treatment substantially increased the mRNA levels of the cardiac transcription factors Gata4, MEF2C and Nkx2.5, and of the contractile genes cTNNT2, MYH7, MHY6 and ACTC1 in W^Cre^CSCs undergoing myogenic differentiation, ensuing into a significant higher number of cTnI positive myocytes in vitro (61 ± 11 vs. 19 ± 5 in control untreated cells, *p* < 0.01) (Figure 6D,E).

**Figure 7:**
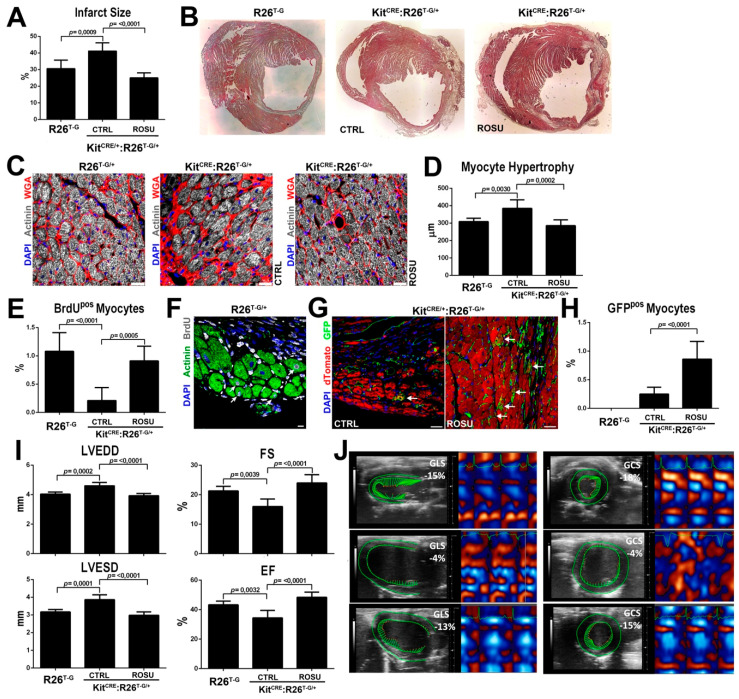
HMG-CoA reductase inhibitors ameliorate in vivo regenerative defect of CSCs from c-kit haploinsufficient mice. (**A**) Bar graphs showing infarct size assessment 28 days after coronary ligation in c-kit wild type control R26^T-G/+^ mice and in ROSU-treated and un-treated (control, CTRL) c-kit^CRE/+:R26T-G/+^ mice. *n* = 6 per group. (**B**) Light microscopy images showing H&E staining of infarcted hearts from c-kit wild type control R26^T-G/+^ and CTRL or ROSU treated c-kit^CRE/+:R26T-G/+^ mice (representative of *n* = 6 mice per group) (**C**) Representative confocal images of cardiac cross-sections showing higher myocyte hypertrophy in the border zone of CTRL-treated c-kit^CRE/+:R26T-G/+^ vs. control R26^T-G/+^ mice 28 days after MI (WGA, wheat germ agglutinin, red). Myocyte hypertrophy is reduced by ROSU in c-kit^CRE/+:R26T-G/+^ mice. Scale bar = 50 µm. (**D**) Bar graphs showing cardiomyocyte size in c-kit wild type control R26^T-G/+^ and untreated (CTRL) and ROSU-treated c-kit^CRE/+:R26T-G/+^ mice. *n* = 6 per group. (**E**) Number of newly-generated BrdU^pos^ myocytes in the border zone of infarcted control R26^T-G/+^ and un-treated (CTRL) and ROSU-treated c-kit^CRE/+:R26T-G/+^ mice 28 days after MI surgery. *n* = 6 per group. (**F**) Representative confocal microscopy image of BrdU incorporation in the border zone of infarcted hearts from control R26^T-G/+^ mice. Scale bar = 10 m. (**G**) Representative confocal microscopy images of GFP^pos^ cardiomyocytes in the border zone of infarcted hearts from un-treated (CTRL) and ROSU treated c-kit^CRE/+:R26T-G/+^ mice. Scale bar = 50 m. (**H**) Bar graph with cumulative data showing the number of GFP^pos^ myocytes in the border zone of CTRL or ROSU-treated c-kit^CRE/+:R26T-G/+^ mice 28 days after surgery. (**I**) Echocardiography assessment of LV function 28 days after MI in control c-kit wild type R26^T-G/+^ mice and CTRL- or ROSU-treated c-kit^CRE/+:R26T-G/+^ mice. *n* = 6 per group. LVEDD = left ventricle end diastolic diameter, LVESD = left ventricle end systolic diameter, EF = ejection fraction, FS = fractional shortening. (**J**) Representative echo images and longitudinal and circumferential strain (GLS and GCS, respectively) traces in long and short axis, respectively, from control c-kit wild type R26^T-G/+^ mice and CTRL- or ROSU-treated c-kit^CRE/+:R26T-G/+^ mice 28 days after MI. ROSU improves myocardial strain (both circumferential and longitudinal) in c-kit^CRE/+:R26T-G/+^ mice to values similar to c-kit wild type control R26^T-G/+^ mice. All data are Mean ± SD.

We and others have shown that c-kit haploinsufficiency as well as c-kit kinase dysfunction in W mice [10,40,41,42] negatively affect cardiac repair after myocardial infarction. Thus, we tested whether HMG-CoA reductase inhibitors rescue the deficit in resident CSC activation and new myocyte formation in W mice [10,13]. To this aim, 12 week-old female c-kit^CRE/+:R26T-G/+^ double heterozygous mice and R26^T-G/+^ heterozygous controls were subjected to myocardial infarction (MI) by left coronary artery permanent ligation followed by systemic BrdU administration through mini-osmotic pumps. c-kit^CRE/+:R26T-G/+^ double heterozygous mice were either treated in the absence or presence of ROSU (20 mg/Kg daily) in drinking water from 14 days before to 28 days after MI. As previously reported, c-kit^CRE/+:R26T-G/+^ double heterozygous mice presented with larger infarcts than R26^T-G/+^ heterozygous controls at 28 days after permanent coronary ligation (Figure 7A,B). 

Accordingly, c-kit^CRE/+:R26T-G/+^ mice show an exaggerated cardiomyocyte hypertrophy when compared to R26^T-G/+^ mice (Figure 7C,D), which was associated with a significant reduced new cardiomyocyte formation as detected by the decreased number of BrdU^pos^ cardiomyocytes in the infarct border zone when compared to control R26^T-G/+^ mice (0.21 ± 0.23% vs. 1.1 ± 0.33%) (Figure 7E,F). ROSU treatment increased the number of CSC-derived new GFP^pos^ cardiomyocytes in the infarct border zone of c-kit^CRE/+:R26T-G/+^ mice (0.86 ± 0.31% vs. 0.25 ± 0.12% untreated c-kit^CRE/+:R26T-G/+^ mice) (Figure 7G,H). The latter was associated with an increased number of BrdU^pos^ cardiomyocytes (0.91 ± 0.26%) by ROSU to a rate comparable with WT mice 28 days after permanent coronary ligation, showing the rescue of c-kit haploinsufficiency-dependent myocyte regeneration defect by ROSU (Figure 7E,F). ROSU treatment also reduced cardiomyocyte hypertrophy in c-kit^CRE/+:R26T-G/+^ mice after MI (Figure 7C,D).

In concordance with the above histology data, the more severe LV dysfunction in c-kit^CRE/+:^R26^T-G/+^ as compared with control R26^T-G/+^ mice assessed by standard echocardiography was rescued by ROSU treatment in vivo (Figure 7I). Interestingly, speckle-tracking based strain analysis on long axis and short axis B-mode further confirmed that ROSU improved myocardial contractility in c-kit^CRE/+:^R26^T-G/+^, as indeed it increased both the global longitudinal and circumpherential strain to values similar to wt mice (Figure 7J).

These data further establish that HMG-CoA reductase inhibitors foster CSC activation, increasing their myogenic potential after myocardial damage in vivo.

## 3. Discussion

The main findings of this study are that HMG-CoA reductase inhibitors promote CSC activation and increase their myogenic specification and differentiation. This class of drugs fosters CSC activation by enhancing their growth, clonogenesis and spherogenesis. Importantly, when CSCs are primed with statins, they show a more robust commitment and specification into cardiomyocytes in vitro. Following treatment with statins in vivo, infarcted rodent hearts show an increased number of CSC-enriched cardiac cells in the infarct border zone and associated with their myogenic commitment produce a significant rise of newly-formed BrdU^pos^ cardiomyocytes.

For decades, it has been advocated and experimentally shown that statins have pleiotropic and cholesterol-independent effects on the cardiovascular system [43]. Among these pleiotropic effects, statins modulate pro-inflammatory cytokine production, ROS generation, endothelial function, cardiac hypertrophy/fibrosis and myocyte death [43]. Yet, while experimental data are well-defined in showing the cardiovascular protective anti-inflammatory effects of statins, which are independent of LDL-cholesterol lowering [24,25,26], the relative contributions of statin pleiotropy (including anti-inflammatory effects, pros-survival, pro-angiogenic, etc.) to clinical outcomes remain a matter of debate [43]. Indeed, the clinical trials testing human monoclonal antibodies to inhibit proprotein convertase subtilisin–kexin type 9 (PCSK9), which do not hit pleiotropic molecular pathways as statins, and thus do not possess pleiotropic effects, show a clinical cardiovascular benefit solely through an unprecedented LDL-C reduction [44]. Nevertheless, several studies have shown that statins could modulate the biological characteristics and function of several embryonic and adult stem and progenitor cells, which is independent from the cholesterol-lowering effect [45]. Indeed, statins modulate self-renewal and differentiation of stem cells [29,45]. Additionally, statins enhance mobilization and homing of bone marrow-derived stem cells, while they also improve engraftment and survival of transplanted stem cells [46,47,48,49]. Altogether, these data postulate that statin treatment could be an effective method to facilitate stem cell therapy [31,45].

The potential beneficial effects of statins on stem/progenitor cells, however, have not been consistently observed for many tissue-specific stem/progenitor cells [50]. Indeed, it has been shown that statins reduce neural stem cell growth and differentiation, where they activate autophagy and apoptosis [50]. Additionally, treatment of mouse embryos with statins acting through the HIPPO pathway interfere with trophectoderm specification, and thereby inhibit blastocyst formation [50]. 

In the present work, we assessed statins effects on rat/mouse CSCs by exposing them to statins from the time of their isolation to mimic more closely the chronic (practically life-long) exposure of cardiovascular patients to these drugs. Using this regime, we show that statins increase CSC growth and self-renewal, as documented by their increased expansion, clonal and sphere formation in vitro. Importantly, the most striking effect of statins were on CSCs commitment to myogenic lineages. CSCs exposed to statins show a pronounced commitment to myogenesis through a balanced and very robust upregulation of myogenic transcription factors and ensuing expression of sarcomeric genes. These effects raise the potential for a similar effect on senescent CSCs in aging, where both CSC growth and differentiation are significantly impaired [3,51,52,53,54,55]. The effects of acute exposure to statins both in vitro and in vivo remains to be shown.

Statins’ molecular mechanism of action on cholesterol metabolism depends mainly on the inhibition of the post-translational prenylation of small GTP-binding proteins such as Rho [56]. On the other hand, activation of Akt represents a mechanism that can account for some of the beneficial effects of statins, including the promotion of new blood vessel growth [57] and increased number of endothelial progenitor cells [23,31] as well as inhibition cell senescence [58]. Accordingly, here, we show that statins also activate CSC expansion and survival through Akt activation. Moreover, they revert the reduced Akt phosphorylation in CSCs from c-kit-haploinsufficient mice, thereby increasing myocyte commitment of these cells both in vitro and in vivo. The statins’ reversal of Akt inhibition in c-kit haploinsufficient cells closely mimics (even though it does not equate to) the functional effects of re-establishing the diploid level of c-kit in rescuing the myogenic potential of CSCs [10].

Tuning the dynamics and the resolution of a post-MI inflammatory process, controlling the hypertrophy of the surviving cardiomyocytes and promoting most efficient replacement of the lost cells remain vital issues for cardiovascular pharmacology. Of interest, our work adds a new piece to the puzzle of the pleiotropic beneficial effects of statins in the post-MI myocardial response by documenting that statins, by acting on the CSCs, also improve the regenerative potential of the adult heart. Nevertheless, the data presented falls short to conclude that CSC activation and ensuing new myocyte formation are pivotal phenomena within the beneficial effects fostered by statins. Indeed, as it has been shown previously that statins target different aspects of pathological remodeling, which are all significant in explaining their positive effects. Both the significant reduction in cardiomyocyte apoptosis (Figure 4F), reflecting an enhanced survival of pre-existing cardiomyocytes and also the significant prevention of reactive maladaptive cardiomyocyte hypertrophy (Figure 4D), indicating a better overall function of pre-existing cardiomyocytes, are key mechanisms in the beneficial effects of statins on cardiac anatomy and function after MI. However, until now, these studies have not considered their possible effect on CSC-dependent myocardial regeneration. As shown here, the clear numerical and functional benefits by statins on CSCs and their myogenic progeny indicates that the process of the activation of resident CSCs is also relevant. It should be pointed out, however, that the data on rat MI (Figure 4) cannot ascertain whether the new myocytes are direct progeny of CSC differentiation and/or whether they arise from division of pre-existing cardiomyocytes. Pre-existing myocyte division has been claimed by some as the origin of new myocytes in the adult, in part based on data obtained with c-kit/Cre mice [4,5,6,7,8,9,59]. Unfortunately, the data obtained with these mice [4,5,6,7,8,9] do not help to resolve the issue about neomyogenesis in the adult heart either in physiological or pathological conditions [60,61]. Because of their c-kit hemizygosity, these mice fail to efficiently recombine and fate-map the CSCs in vivo and, additionally, produces a significant regenerative defect on CSCs [10]. Independently of their origin, it is clear that statins increase the number of new myocytes after MI in both rats and mice (Figure 5 and Figure 7), which correlates with the increased number and myogenicity of the CSCs. Because of their c-kit hemizygosity, failing to quantitatively and qualitatively track the progeny of the CSCs, the present data in these mice only show that statins improve the inherent regenerative defects of CSCs (Figure 6) and they also increase the number of new myocytes post-MI [Figure 7] in these mice. Therefore, these data cannot correctly quantify the contribution of the CSCs nor exclude the contribution, if any, of pre-existing myocyte division, to the statins-induced increase in post-MI neomyogenesis. Novel and efficient c-kit/Cre mice are needed to definitively address this outstanding question.

Although there are some differences between individual statins in their effect on the lipid profile [62], their clinical benefits are generally reproducible and their benefit is commonly regarded as a class effect [62,63]. As shown here, both hydrophilic (pravastatin and rosuvastatin) and lipophilic (simvastatin) statins foster CSC activation and myogenic specification in vitro. For this reason, we only tested rosuvastatin in the rodent MI model and show that this statin increases CSC number and activation with resulting new myocyte formation, which contributes to myocardium remodeling. Considering the in vitro data, it is reasonable to expect similar benefits from the other statins, particularly when both pravastatin and simvastatin have been reproducibly reported to ameliorate myocardial remodeling after myocardial infarction in both rats and mice [46,64,65]. Yet, the in vivo effects of other statins on CSC and myocyte refreshment and whether these effects are extrapolatable to humans remain to be demonstrated.

## 4. Materials and Methods

### 4.1. Animals

All animal experimental procedures were approved by Magna Graecia Institutional Review Boards on Animal Use and Welfare and performed according to the Guide for the Care and Use of Laboratory Animals from directive 2010/63/EU of the European Parliament. All animals received humane care, and all efforts were made to minimize animal suffering. Mice and rats were housed under controlled conditions of 25 °C, 50% relative humidity and a 12 h light (6:00–18:00) and 12 h dark cycle, with water and food (containing 18.5% protein) available ad libitum. Mice and rat were anesthetized by intraperitoneal injections of Tiletamine/Zolazepam (80 mg/Kg) or inhaled isoflurane (isoflurane 1.5% oxygen 98.5%, Iso-Vet, Healthcare).

As wild type animals, C57BL/6J mice were used (Jackson Labs, stock number 000664). Wistar rats were purchased from Charles River Laboratories. Constitutive c-kit^CreGFPnls/+^ (above abbreviated as c-kit^CRE/+^) [4] were provided by Dr. Jeffrey D. Molkentin (Cincinnati Children’s Hospital Medical Center, Cincinnati, OH) through an MTA and payment of a fee for preparation and distribution costs. Heterozygous Kit^CreER(T2)/+^ mice were a generous gift by Dr. Dieter Saur [66]. Heterozygous c-kit^CRE/+^ were crossed with homozygous Gt(ROSA)26Sortm4(ACTB-tdTomato,-EGFP)Luo/J Cre-reporter mice (abbreviated above as R26^T/G/+^, Jackson Labs, stock number 007576) to generate double heterozygous animals for experimental purposes. The *n* value for each experimental group is specified in the relative figure legends.

### 4.2. Cell Reagents

To test whether HMG-CoA reductase inhibitors regulate myocardial remodeling and proliferation and differentiation of resident CSCs in vitro, three different statins were used: simvastatin, pravastatin and rosuvastatin, all of which were from Sigma Aldrich. For in vitro treatment, chemical compounds were prepared by dissolving the powder with dimethylsulfoxide (DMSO). For in vivo study the powder was directly dissolved in drinking water. For Akt inhibition, MK2206 (Enzo Biochem, New York, NY, USA) was used at a concentration of 1 μM, a dose previously established not to have cytotoxic effects on CSCs [36].

### 4.3. CSC Isolation and Culture

Cardiac stem cells (CSCs) were isolated from adult mouse and rat hearts by enzymatic dissociation using a Langerdoff-modified apparatus or using gentleMACS Dissociator (Miltenyi Biotec) as previously reported [11,12,13,33]. Myocyte-depleted cardiac small cells were used to obtain CSC-enriched CD45^neg^CD31^neg^c-kit^pos^ cells by FACS sorting with specific fluorochrome-conjugated antibodies or by MACS technology with direct CD45 and CD31 negative and then c-kit positive specific anti-mouse microbeads sorting (Miltenyi Biotec) as previously reported [11,12,13,33]. Freshly isolated and cloned CSC-enriched CD45^neg^CD31^neg^c-kit^pos^ cardiac cells derived from mouse or rat hearts were plated in gelatin-coated dishes in CSC growth medium containing DMEM-F12 Ham’s (Gibco, Life Technologies) with insulin-transferrin-selenium (1%, Life Technologies), epidermal growth factor (final medium concentration: 20 ng/mL, Peprotech), basal fibroblast growth factor (final medium concentration: 10 ng/mL, Peprotech) and leukemia inhibitory factor (final medium concentration: 10 ng/mL, Miltenyi) and a 1:1 ratio of Neurobasal medium (Gibco, Life Technologies) containing 37 mg of L-glutamine, B27 supplement (1X, Life Technologies) and N2 supplement (1X, Life Technologies), penicillin-streptomycin (1%, Life Technologies), Fungizone (0.1%, Life Technologies) and gentamicin (0.1%, Life Technologies). The CSC growth medium was sterilized through a 0.22-µm pore filter into a sterile container and stored at 4 °C. The CSC growth medium was supplemented with 10% ESQ-FBS (Life Technologies). Cells were maintained at 37 °C in ambient O_2_ (21%) and 5% CO_2_. Media were replenished every 48 h and cells were passaged at a 1:4 ratio.

### 4.4. CSC Clonogenic and Spherogenesis Assay In Vitro

Single cell cloning was employed, depositing single mouse or rat CD45^neg^CD31^neg^c-kit^pos^ CSCs into 96-well gelatin-coated Terasaki plates by serial dilution and were grown in CSC growth medium in presence or absence of HMG-CoA reductase inhibitors (ROSU, 1 µM, SIM, 1µM or PRAVA, 5 µM). After 2 weeks clones were identified and counted. Clonogenicity was determined by counting the number of clones in each 96-well plate and expressed as a percentage of plated single cells. A total of at least 10 plates were analyzed for each experiment. For cardiospheres generation, 1 × 10^5^ mouse-derived CSCs were placed in bacteriological dishes with CSC growth medium in presence or absence of HMG-CoA reductase inhibitors (ROSU, 1 µM, SIM, 1 µM or PRAVA, 5 µM). Rat cardiospheres were generated by placing 1 × 10^5^ cells in bacteriological dishes with cardiosphere generation medium (mCSFM), composed of 1:1 ratio of CSC growth medium and Neuro Basal Media supplemented with B27 and N2 supplements (Life Tech) as previously reported [12]. Rat cardiosphere were generated in presence or absence of HMG-CoA reductase inhibitors (ROSU, 1 µM, SIM, 1 µM or PRAVA, 5 µM). Cardiospheres were counted per plate at 14 days and the number expressed as a percentage relative to the number of plated CSCs [12].

### 4.5. Cardiac Differentiation Potential and Cardiosphere Myogenic Differentiation Assay In Vitro

Mouse- or rat-derived CSCs were placed in CSC growth medium in bacteriological dishes for 5–7 days in presence or absence of HMG-CoA reductase inhibitors (ROSU, 1 µM, SIM, 1 µM, or PRAVA, 5 µM) for cardiospheres generation.

Rat cardiospheres were then switched in LIF-deprived basic differentiation medium consisting of α-MEM, dexamethasone (1 µM), ascorbic acid (50 μg/mL) and β-glycerophosphate (10 mM) (all from Sigma) with 3% FBS (Invitrogen). For specific myocyte cell commitment, TGF-β1 (5 ng/mL, Peprotech) was added to the medium and complete differentiation media was refreshed every 72 h. Cell differentiation was evaluated at 14 days as previously described [12].

Mouse cardiospheres were switched to base differentiation medium consisting of StemPro^®^-34 SFM (a serum-free medium conditioned with StemPro^®^-Nutrient Supplement, Gibco, Life Technologies), Glutamine (2 mM) and penicillin-streptomycin (1%, Life Technologies). For specific myocyte differentiation BMP4 (10 ng/mL, Peprotech), Activin-A (10 ng/mL first day and then 5 ng/mL, Peprotech), β-FGF (10 ng/mL, Peprotech), Wnt-11 (150 ng/mL, R&D System) and Wnt-5a (150 ng/mL, R&D System) were added to base differentiation medium from day 0 to day 4. At day 4, differentiating cardiospheres were pelleted and transferred to laminin (1 µg/mL) coated dishes and Dkk-1 (150 ng/mL, R&D System) was added to base differentiation medium from day 5 to day 14. Differentiated cardiospheres were either trypsinized for RNA isolation or fixed with 4% Paraformaldehyde (PFA, Sigma #P6148) and stained for cTnI. For the latter, nuclei were counterstained with the DNA binding dye, 4,6-diamidino-2-phenylindole (DAPI, Sigma) at 1 µg/mL. Cells were evaluated using confocal microscopy.

### 4.6. Proliferation and Apoptosis Assay In Vitro

CSC proliferation was evaluated through BrdU incorporation assay and the growth curve, at the indicated time points, on mouse- and rat-derived CSCs. CSCs were plated at density of 1 or 2 × 10^4^ in 12- or 6-well dishes and then serum starved for 48 h. After this time, starvation medium was replaced by CSC growth media and then cells were then treated with rosuvastatin or simvastatin (1 µM) or pravastatin (5 µM) or just vehicle and BrdU (10 µM, Roche) was added to the medium every 12 h. BrdU incorporation was measured after 24 h by immunostaining using the BrdU detection system kit (Roche). Nuclei were stained with DAPI. Cells were evaluated using fluorescent microscopy.

Growth curve assay was archived by planting 2 × 10^4^ cells in 60 mm gelatin-coated dishes in CSC growth medium and then serum starved for 48 h when starvation medium was replaced by CSC growth media and cells were treated with rosuvastatin or simvastatin (1 µM) or pravastatin (5 µM) or just vehicle. Cells were then trypsinized and counted using trypan blue, 1:1 ratio, at the indicated time points.

CSC apoptosis was detected using the terminal deoxynucleotide transferase-mediated dUTP nick-end labelling (TUNEL) assay 12 h after H_2_O_2_ damage in presence or absence of the indicated statins or Akt inhibitor (MK2206) [18].

### 4.7. Immunocytochemistry

For myogenic differentiation, cardiospheres derived from CSCs were cultured on glass chamber slides for 14 days and, after fixation with 4% PFA for 20 min on ice, stained with a rabbit anti-cTnI (1:50 dilution, Santa Cruz). Cells were incubated with an anti-rabbit secondary antibody (1:100 dilution, Jackson Immunoresearch). Nuclei were stained with DAPI. Fluorescence was visualized and images acquired with confocal microscopy (LEICA TCS SP8).

### 4.8. Quantitative RT-PCR (qRT-PCR)

RNA was extracted from clonogenic mouse- and rat-derived CSCs using TRIzol Reagent (Ambion) and quantified using a Nanodrop 2000 Spectrophotometer (Thermo Fisher Scientific). Reverse transcription was performed with 0.5–1 µg of RNA using the High Capacity cDNA Kit (Applied Biosystems). Quantitative RT-PCR was performed using TaqMan Primer/Probe sets (Applied Biosystems) using StepOne Plus real Time PCR System (Applied Biosystems). The following genes were tested: Kit, GATA-4, Nkx2.5, MEF2c, MHC6, MHC7, TNNT2, MYL2 and ACTC1 (see Table 1). Data were processed by the ΔCt method using StepOne Software v2.3 and mRNA was normalized to the housekeeping gene, GAPDH. Heatmaps illustrating CSC myogenic differentiation were then created in color scales representing fold changes over baseline value for each gene. All reactions were carried out in technical triplicate.

### 4.9. Western Blot Analysis

Immunoblots were carried out using protein lysates obtained from mouse- and rat-derived CSCs. Generally, aliquots equivalent of ~40 to 70 µg of proteins were separated on gradient (6–15%) SDS-polyacrylamide gels. After electrophoresis, proteins were transferred onto nitrocellulose filters, blocked with either 5% dry milk or 5% bovine serum albumin, and incubated with Ab against Akt (1:1000, Cell Signaling) and pAkt (1:1000, Cell Signaling) (see Table 2). Proteins were detected by chemiluminescence using horseradish peroxidase-conjugated 2Abs and placing the nitrocellulose filters on a photographic film. The acquisition was archived using Medical X-ray processor 2000 (CARESTREAM). Densitometry was obtained using ImageJ software. Immunoblots were performed in biological quadruplicates and technical duplicates.

### 4.10. Myocardial Infarction Procedure

Myocardial infarction procedure was performed on female Wistar rats (270±20g), 14/16-week-old female R26^T/G/+^ heterozygous mice and 14/16 week-old female c-kit^CRE/+:R26T/G/+^ double heterozygous mice (body weight of 25–30 g), through permanent ligation of the left anterior descending (LAD) coronary artery [10,12]. Animals were intubated with a 22 G tube, ventilated with a mechanical ventilator (28026 mouse ventilator, Ugo Basile, Italy; tidal volume 0.2 mL, 120 strokes/min) and anesthetized with inhaled isoflurane, while kept at 37 ± 2 °C body temperature. The LAD coronary artery occlusion was performed as previously reported [10,12]. The day after MI, all employed mice and rats were subcutaneously implanted with mini-osmotic pumps (ALZET) to systemically release BrdU (50 mg/Kg/day, Sigma B9285) prepared by dissolving the BrdU powder in 50% de-ionized water and 50% DMSO. All the employed animals were divided in different groups on the basis of the experimental arm to receive vehicle or HMG-CoA reductase inhibitors treatment (Rosuvastatin 20 mg/Kg daily) in drinking water for 14 days before and 28 days after LAD ligation. All animals were sacrificed at 28 days after MI and the hearts were fixed in 10% formalin or 4% PFA for immunohistochemistry analysis. Acute mortality rate within 24 h after MI procedure was overall of ~25/30%. The final *n* value for each experimental group is specified in the relative figure legends.

### 4.11. Echocardiography

Prior to echocardiography, mice and rats were anesthetized with isoflurane. Unconscious mice and rats were weighed and secured in a supine position on a temperature-controlled restraining board. Anesthesia was maintained with 1–2% isoflurane in oxygen delivered through a nose cone. All hair in the thoracic region was removed using a depilatory agent, and the area was cleaned with water. Echocardiographic images were obtained with a Vevo 3100 system (Visualsonics, Inc.) as previously described [10,12,13]. Briefly, parasternal long and short axis views were obtained with both M-mode and two-dimensional echocardiography. LV dimensions (LV end diastolic diameter, LVEDD and LV end systolic diameter, LVESD) were measured perpendicular to the long axis of the ventricle at the mid-chordal level on three consecutive cycles and averaged by two independent observers (J.S. and M.A.) in a blinded fashion. Fractional shortening and LV ejection fraction were accordingly calculated. Advanced cardiac analysis (regional and global cardiac measurements) was assessed by speckle-tracking echocardiography (Vevo LAB analysis software; VisualSonics). Image analysis was performed according to manufacturer’s instructions as previously reported [10,13]. The *n* value for each experimental group is specified in the relative figure legends.

### 4.12. Tissue Harvesting, Histology and Immunohistochemistry

For immunohistochemistry analysis, hearts derived from the relative mice and rats were isolated as previously reported [10,12,67,68,69,70]. Hearts were fixed with 10% buffered formalin or with 4% PFA and embedded in paraffin or in Optimal Cutting Temperature Compound (OCT), respectively. Cardiomyocyte cross-sectional area was measured through immunostaining with Wheat Germ Agglutinin Alexa Fluor 594 or 488 conjugate, WGA (1:200 dilution; Invitrogen) and digital analysis of acquired cardiac cross-section images (Leica, 1128 LAS AF Software). Cardiomyocyte diameter was measured across the nucleus on three transverse sections (~500 myocytes/animal were sampled). For BrdU detection, antigen retrieval was achieved using Target Retrieval Solution, Citrate pH 6 (DAKO). The following primary antibodies were used: anti-c-kit (1:50 dilution; R&D Systems), anti-Sarcomeric Actinin (1:50 dilution; Santa Cruz), anti-Cardiac Troponin I (1:50 dilution; Santa Cruz), monoclonal antibody against cardiac myosin (MF 20, DSHB), anti-Actinin (1:50 dilution; Santa Cruz), anti-GFP (1:50 dilution; Rockland), anti-Tdt (1:50 dilution; Roche), anti-BrdU (1:50 dilution; Roche) and GATA-4 (1:50, dilution; Santa Cruz) (see Table 2). The primary antibody was revealed by respective anti-mouse IgG, anti-rabbit IgG or anti-goat IgG secondary antibody (1:100 dilution; Jackson Immunoresearch). The nuclei were counterstained with DAPI. To evaluate the cardiomyocyte progeny of GFP-positive CSCs in vivo, the number of double positive GFP^pos^/Troponin^pos^ cardiomyocytes were counted in cardiac cross sections from sham operated- and MI-subjected mice for each power field using a ×63 objective for a total of 20 fields [11]. Specifically, the border infarct zone of each mid and apical region was analyzed for the MI studies. The number GFP^pos^ cardiomyocytes was expressed as a percent fraction of the total cardiomyocyte number per mm^2^ [10,11]. Accordingly, the number of BrdU^pos^ cardiomyocytes was expressed as a percent fraction of the total cardiomyocyte nuclei as previously reported [11]. All stainings were acquired and analyzed using confocal microscopy (LEICA TCS SP8).

### 4.13. Statistical Analysis

Statistical analysis was performed with GraphPad Prism version 6.00 for Macintosh (GraphPad Software). Quantitative data are reported as mean ± SD and binary data by counts. Significance between two groups was determined by Student’s t test or paired t test as appropriate. For comparison between multiple groups, ANOVA was used. A *p* value <0.05 was considered significant. Bonferroni post-hoc method was used to locate the differences. In these cases, the Type 1 error (α = 0.05) was corrected by the number of statistical comparisons performed. For the in vitro cell and molecular biology experiments with an *n* = 4 sample size, given the low number of the sample, the Kruskal–Wallis test (for multiple-group comparison) and the Mann–Whitney U test (for comparison between two groups) were performed.

## 5. Conclusions

In conclusions, the present study demonstrates that HMG-CoA reductase inhibitors activate the regenerative potential of CSCs in vitro by both fostering their expansion and myogenic differentiation through the activation of the serine/threonine kinase Akt. Importantly, statins increase CSC activation in vivo after myocardial infarction in rodents enhancing CSC-derived new cardiomyocyte formation, contributing to the improved cardiac remodeling after ischemic insult by this class of drugs.

## Figures and Tables

**Table 1:** List of RT-PCR primers.

GENE	SPECIES	ID NUMBER
Gapdh	Mouse	Mm99999915_g1
Mef2c	Mouse	Mm01340842_m1
Nkx2.5	Mouse	Mm01309813_s1
Gata4	Mouse	Mm00484689_m1
c-kit	Mouse	Mm00445212_m1
Myh7	Mouse	Mm01319006_g1
Myl2	Mouse	Mm00440384_m1
Actc1	Mouse	Mm01333821_m1
TnnT2	Mouse	Mm01290256_m1
Gapdh	Rat	Rn01775763_g1
Gata4	Rat	Rn01530459_m1
Mef2c	Rat	Rn01494046_m1
Tnnt2	Rat	Rn01483694_m1
Myh7	Rat	Rn01488777_g1
Myo6	Rat	Rn01521319_m1
Actc1	Rat	Rn01513700_g1

**Table 2:** List of antibodies.

ANTIGEN	ANTIBODY ID	COMPANY	APPLICATION ^1^
AKT	9272	Cell Signaling	WB
pAKT	4058S	Cell Signaling	WB
WGA		Invitrogen	IH
MF20	Ab_2147781	DSHB	IH
c-kit	AF 1356	R&D System	IH
GATA4	sc-9053	SantaCruz Biotech	IH
GFP		Rockland Immunochemicals	IH
α-Actinin	sc-17809	Santa Cruz Biotech	IH
cTNI	H170	Santa Cruz Biotech	IH
BrdU		Roche	IH
α-SARC	A2172	SIGMA	IH
TdT	9272	Roche	IH

^1^ IH denotes Immunohistochemistry; WB denotes western blot.

## Abbreviations

CSCs	Cardiac Stem Cells
MI	Myocardial Infarction
HMG-CoA	3-hydroxy-3-methyl-glutaryl-coenzyme
LV	Left ventricle
